# Fabrication and Enhanced Thermal Conductivity of Boron Nitride and Polyarylene Ether Nitrile Hybrids

**DOI:** 10.3390/polym11081340

**Published:** 2019-08-13

**Authors:** Ling Tu, Qian Xiao, Renbo Wei, Xiaobo Liu

**Affiliations:** Research Branch of Advanced Functional Materials, School of Materials and Energy, University of Electronic Science and Technology of China, Chengdu 611731, China

**Keywords:** thermal resistance, thermal conductivity, boron nitride, hybrid

## Abstract

Excellent thermal resistance and thermal conductivity are preconditions of materials to be used at elevated temperatures. Herein, boron nitride and polyarylene ether nitrile hybrids (PEN-g-BN) with excellent thermal resistance and thermal conductivity are fabricated. Phthalonitrile-modified BN (BN-CN) is prepared by reacting hydroxylated BN with isophorone diisocyanate (IPDI) and 3-aminophxylphthalonitrile (3-APN), and then characterized by FT-IR, UV-Vis, and X-ray photoelectron spectroscopy (XPS). The obtained BN-CN is introduced to a phthalonitrile end-capped PEN (PEN-Ph) matrix to prepare BN-CN/PEN composites. After curing at 340 °C for 4 h, PEN-g-BN hybrids are fabricated by a self-crosslinking reaction of cyano groups (-CN) from BN-CN and PEN-Ph. The fabricated PEN-g-BN hybrids are confirmed through FT-IR, UV-Vis, SEM and gel content measurements. The PEN-g-BN hybrids demonstrate excellent thermal resistance with their glass transition temperature (*T*_g_) and decomposition temperatures (*T*_d_) being higher than 235 °C and 530 °C, respectively. Additionally, the thermal conductivity of the prepared PEN-g-BN hybrids is up to 0.74 W/(m·k), intensifying competitiveness of PEN-g-BN hybrids for applications at elevated temperatures.

## 1. Introduction

Thermal conductive materials, which are indispensable functional materials in modern industry and life, are widely used in aerospace, electromagnetic shielding, microelectronic packaging, and other fields [1,2,3,4]. More specifically, with the development of electronic components towards miniaturization and high integration, the preparation of electronic packaging materials with excellent thermal stability and thermal conductivity is one of the focuses of research [5,6]. Polyarylene ether nitrile (PEN), as a special kind of polymer engineering material, is widely used in the aerospace, automobile manufacturing, and electronic industry fields due to its excellent thermal stability, corrosion resistance, and mechanical properties [7,8,9]. In addition, since the PEN backbone contains a large number of cyano groups (-CN), it shows the following advantages: (1) the -CN exhibits a strong polarity which can promote the adsorption of the polymer on various placodes and enhance the dielectric constant of PEN; (2) the -CN at the molecular chain of PEN self-crosslinks at elevated temperature, thereby increasing the application temperature of PEN; (3) the -CN of PEN can react with other functional fillers, improving the interfacial compatibility between the matrix and the incorporated fillers [10,11,12,13]. However, like most polymer resins, the relatively poor heat dissipation and low thermal conductivity of PEN restrict its further application in areas like electronic packaging and heat exchange engineering, etc. [12].

According to heat transfer theory, heat is transmitted by free electrons or lattice vibration in solid matter. However, there are no free electrons in PEN, and PEN demonstrates poor crystallization, resulting in poor thermal conductivity. Therefore, for the sake of enhancing its thermal conductivity, an effective way is to introduce fillers with free electrons or a regular lattice forming a thermal conductive channel in the PEN matrix. The addition of fillers with free electrons (such as Au, Ag, carbon nanotube (CNT), or graphene oxide (GO), etc.) also leads to a rapid increase in the conductivity of the system, making the resulted composite unsuitable for use in the field of electronic packaging [14,15,16]. The only remaining option is adding highly thermally conductive fillers with regular crystals (such as Al_2_O_3_, AlN, and BN) [17,18,19]. Among these highly thermally conductive fillers, hexagonal boron nitride (h-BN) has been widely used owing to its advantages, which include unique electrical insulating properties, an ultra-low thermal expansion coefficient, excellent thermal conductivity, and chemical stability [20,21,22,23]. In addition, h-BN has a layered structure similar to graphite and the thermal conductivity of its 001 plane is as high as 300 W/(m·k) [24,25,26]. However, like most inorganic fillers, h-BN shows poor compatibility with PEN, which limits the further enhancement of thermal conductivity of the resulting composite materials.

To further improve the thermal conductivity of PEN, chemical and/or physical modification of h-BN is essential [27,28,29]. Xiao et al. [12] obtained about two atomic layers of BN nanosheets (BNNS) by ultrasonic peeling and then introduced them into PEN to obtain a composite with thermal conductivity of 0.45 W/(m·k). On the one hand, the high thermal conductivity is derived from the nanosheet BNNS, which is easier to vibrate (it has been reported that the thermal conductivity of BNNS can be as high as 700 W/(m·k) [30,31]); on the other hand, at the same mass of BN, the volume of BNNS is much larger than that of unstripped h-BN, so it is easier to form heat conduction channels in the composite. However, the time-consuming and low efficiency of fabrication of BNNS is not suitable for large-scale applications. In order to improve the preparation efficiency, Xiao et al. [32] have also proposed preparing BN@(sulfonated PEN (SPEN)) by coating h-BN with SPEN and then adding BN@SPEN to PEN, obtaining a composite with thermal conductivity of up to 0.7 W/(m·k). This method is used to obtain a core-shell structure of fillers by reacting carboxyl groups from SPEN with hydroxyl groups from BN. The similar structure and excellent compatibility between SPEN and PEN has resulted in the homogeneous dispersion of BN@SPEN in the PEN matrix, which improves the thermal conductivity of the system effectively. However, SPEN still has an interface with PEN, which will become larger and larger after a long time of application, thus reducing the comprehensive properties of the composites.

Herein, we report the preparation and improved thermal conductivity of BN and PEN hybrids (PEN-g-BN), which are connected by a covalent bond in this research. Phthalonitrile-modified BN (BN-CN) is prepared by a well-established method with reactants of BN-OH, isophorone diisocyanate (IPDI), and 3-aminophxylphthalonitrile (3-APN). The BN-CN is incorporated into a matrix of phthalonitrile end-capped PEN (PEN-Ph) and then reacted with PEN-Ph at high temperatures, offering hybrids through the self-crosslinking of -CN in the system. Due to the formation of the hybrids, BN demonstrates excellent compatibility with the PEN matrix and improved thermal conductivity of the hybrids is obtained. 

## 2. Experimental Section

### 2.1. Materials

**Figure 1:**
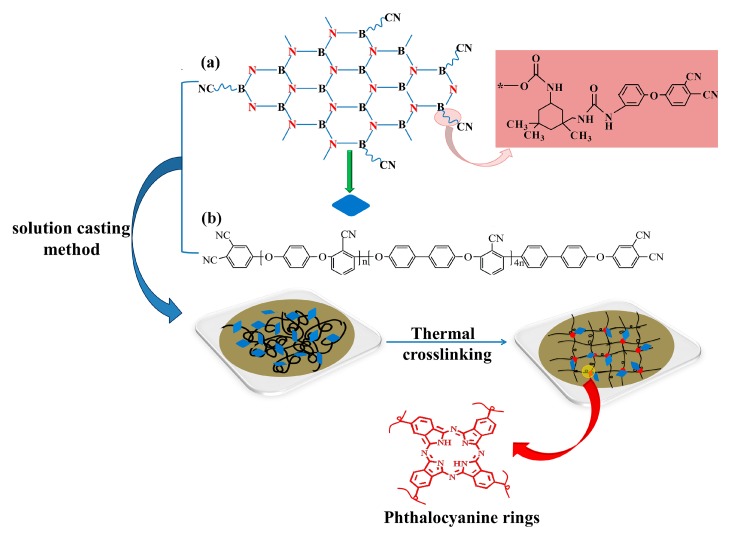
The synthetic route for the preparation of boron nitride and polyarylene ether nitrile hybrids (PEN-g-BN): (**a**) structure of phthalonitrile-modified BN (BN-CN); (**b**) structure of phthalonitrile end-capped PEN (PEN-Ph).

Potassium carbonate (K_2_CO_3_), hydroquinone (HQ), biphenyl (BP), and 2,6-dichlorobenzonitrile (DCBN) were obtained via Chengdu Haihong Chemicals. N-methylpyrrolidone (NMP), acetone, toluene, sodium hydroxide, and hydrochloric acid were acquired from Chengdu Kelong Chemicals. Hexagonal boron nitride was purchased from J&K Chemicals. 3-APN was prepared in our lab, with the detailed preparation process having been reported in the literature [33]. Phthalonitrile-grafted BN was prepared according to a previous work and its structure is shown in Figure 1a [34]. PEN-Ph was synthesized according to previous reports in our laboratory and its structure is shown in Figure 1b [35]. 

### 2.2. Preparation of PEN-g-BN Hybrids

PEN-g-BN hybrids were fabricated through the procedures shown in Figure 1. First, BN-CN/PEN-Ph composites were prepared. BN-CN was gradually added into a 100 mL flask with 5 mL NMP. The mixture was mechanically stirred with violent ultrasonic treatment for 2 h, forming a homogeneous light-brown dispersion. Meanwhile, PEN-Ph was dissolved in 10 mL NMP in another flask. Afterwards, the BN-CN dispersion was mixed with the PEN solution. After homogeneously heating at 200 °C for 0.5 h, the mixture was cast on a glass plate. The glass plate, together with the mixture of the BN-CN dispersion and PEN-Ph solution, were dried within an oven at temperatures of 90, 110, 130, 150, and 170 °C each for 1 h and 200 °C for 2 h. Through cooling back to room temperature and peeling off from the glass substrate, BN-CN/PEN-Ph composite films were obtained. BN-CN/PEN-Ph composites with different loadings of BN-CN (0, 4, 8, 12, and 16 wt %, respectively) were fabricated by changing the weight ratio of BN-CN and PEN-Ph.

The second step is the preparation of PEN-g-BN hybrids. After the fabrication of the BN-CN/PEN-Ph composites, the composites were cured at high temperatures, offering hybrids (PEN-g-BN) through the self-crosslinking of -CN in the system. The best curing procedure is 340 °C for 4 h, according to our laboratory’s previous work [11,36]. 

### 2.3. Characterization

BN-CN was characterized by Fourier transform infrared spectroscopy (200SXV, Nicolet, Madison, WI, USA) with a resolution of 1.0 cm^−1^, UV-Vis absorption spectra (UV2501-PC, Shimadzu, Kyoto, Japan) and X-ray photoelectron spectroscopy (XPS, ESCA 2000, Micro Tech. Co., Kilmarnock, UK). Differential scanning calorimetry (DSC) was performed at TA Instruments DSC-Q100 (TA, New Castle, DE, USA) at a heating rate of 10 °C/min and a nitrogen flow rate of 50 mL/min from room temperature to 320 °C. Thermogravimetric analysis (TGA) measurement was measured with the TGA-Q50 (TA, New Castle, DE, USA) under a N_2_ atmosphere. The gel contents of the PEN-g-BN hybrids were measured by Soxhlet extraction using NMP as a solvent. The mechanical properties of the PEN-g-BN hybrids with a sample size of 150 mm × 10 mm × 40~50 μm were evaluated using a SANS CMT6104 (Shenzhen Shijitianyuan, China) Series Desktop Electromechanical Universal Testing Machine at a stretching rate of 5 mm/min. The results were recorded as the average value for every five samples. Dielectric properties of the PEN-g-BN hybrids at different frequencies and at varying temperatures were characterized by a TH 2819A precision LCR meter (Tong Hui Electronic Co. Ltd., Changzhou, China). The PEN-g-BN hybrids’ micro-morphologies were observed through SEM (JSM-5900LV, JEOL, Tokyo, Japan) operating at 20 kV. The coefficient of thermal expansion (CTE) and cross-linking density of the PEN-g-BN hybrids were measured using a dynamic mechanical analyzer (DMA, QDMA-800, TA, DE, USA) in extension mode over a temperature range from 45 to 340 °C at a heating rate of 5 °C/min under air atmosphere with a force of 0.01 N. The thermal conductivity of the samples was measured with a LFA 457 Laser Flash Apparatus (Netzsch, Selb, Germany). The samples used for the thermal conductivity test were cast into disks with a diameter of about 12.7 mm and a thickness of 1 mm. 

## 3. Results and Discussion

**Figure 2:**
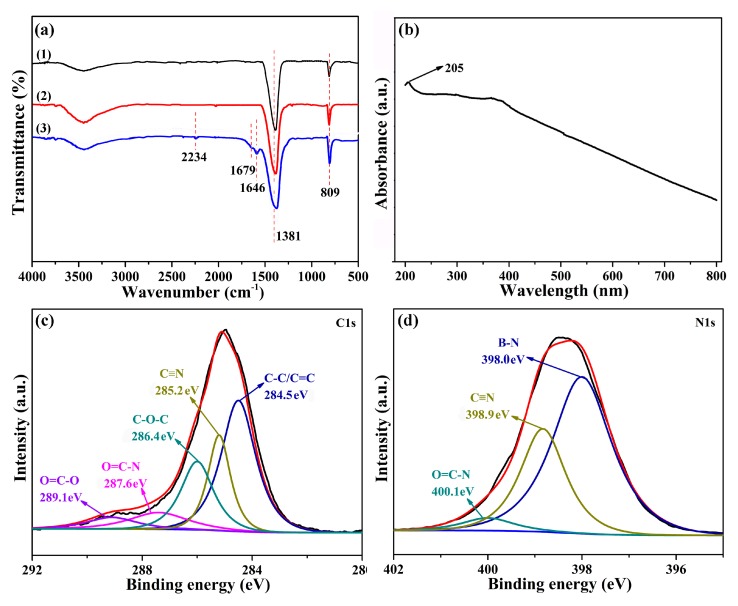
(**a**) FT-IR spectrum; (**b**) UV-Vis absorption spectrum; (**c**) X-ray photoelectron spectroscopy (XPS) C1s spectrum and (**d**) XPS N1s spectrum of BN-CN.

In this paper, BN and PEN hybrids connected by a covalent bond were prepared to improve the thermal conductivity of PEN. Phthalonitrile modified BN (Figure 1a) was prepared by a well-established method, as recorded in a previous report [35]. First, the surface of BN was hydroxylated with a 2 M NaOH solution under a ball-milling procedure to obtain BN-OH. Then, BN-OH was reacted with IPDI, offering BN with an isocyanate group. Finally, BN-CN was prepared by the reaction of isocyanate groups on the BN surface with amino groups from 3-APN. Figure 2a shows the FT-IR spectra of BN, BN-OH, and BN-CN. According to the literature, the strong peaks at 809 and 1381 cm^−1^ result from the stretching vibration and bending vibration of B-N. A peak at around 3500 cm^−1^ originating from –OH is observed from all these samples. Upon normalizing the intensity of the 1381 cm^−1^ peak, the stronger intensity of the –OH peak of BN-OH than that of BN indicates the formation of –OH during the ball-milling process, while the weaker intensity of the –OH peak of BN-CN than that of BN-OH suggests that some of the –OH are consumed after the reaction with IPDI. In addition, the formation of BN-CN can be certified by the peak at 2234 cm^−1^ from the FT-IR spectrum of BN-CN coming from the absorption peak of the cyano group (–C≡N). Furthermore, the absorption peak at 1679 cm^−1^ can be attributed to the carbamate esters, which proves that BN-CN has been successfully prepared. Figure 2b exhibits a UV-Vis absorption spectrum of BN-CN. The characteristic absorption band of BN is confirmed from the absorption peak at 205 nm. Figure 2c,d are the XPS spectra of C1s and N1s of BN-CN, respectively. As can be observed from Figure 2c, the C1s spectrum of BN-CN can be quantitatively differentiated into five different carbon species (C–C/C = C, C≡N, C-O-C, O = C–N, O = C–O). Specially, the peak at 285.4 eV which appears on the BN-CN C1s spectrum corresponds to C≡N, confirming the successful reaction between BN-OH and IPDI/3-APN. Figure 2d shows peaks at 400.1 (C≡N) and 398.9 eV (CONH) on the BN-CN N1s spectrum, proving that BN-CN has been successfully prepared [35]. 

**Figure 3:**
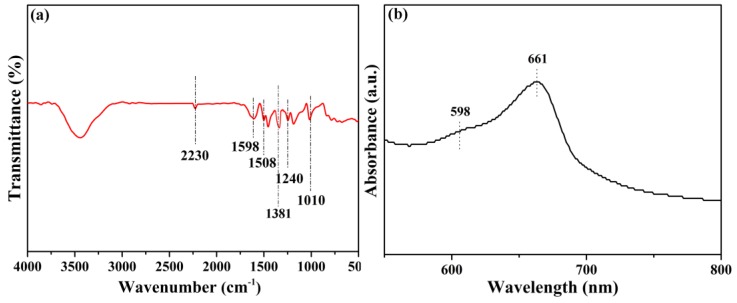
FT-IR spectrum (**a**) and UV-Vis absorption spectrum (**b**) of PEN-g-BN16.

The obtained BN-CN was incorporated into a matrix of phthalonitrile end-capped PEN (Figure 1b) via the solution casting method, offering a BN-CN/PEN composite. Then, the BN-CN/PEN composite was further cured at 340 °C for 4 h to produce the PEN-g-BN hybrid. In this paper, PEN-g-BN (PEN-g-BN0, PEN-g-BN4, PEN-g-BN8, PEN-g-BN12, and PEN-g-BN16) hybrids with BN-CN content of 0, 4, 8, 12, and 16 wt %, respectively, were prepared by changing the content of BN-CN during the fabrication process. The formed PEN-g-BN hybrids were able to be proved by infrared spectroscopy, ultraviolet spectroscopy, and gel content, as shown in Figure 3 and Table 1. The obvious absorption bands which appear at 1240 as well as 1010 cm^−1^ as seen in Figure 3a can be attributed to stretching vibrations of formed phthalocyanine rings. Moreover, the UV-Vis spectrum absorption bands at 661 and 598 nm can be seen from Figure 3b, which further certifies formation of the phthalocyanine rings. In addition, the gel content can be seen to be higher than 90% (Table 1), which is much higher than the content of BN-CN incorporated (lower than 16), indicating the formation of a crosslinked system. Moreover, the cross-linking density can be observed as 0.567, 0.635, 0.744, 0.795, and 0.801 mol/cm^3^ for PEN-g-BN0, PEN-g-BN4, PEN-g-BN8, PEN-g-BN12, and PEN-g-BN16, respectively, according to the DMA results. These results suggest that the PEN-g-BN hybrids were able to be successfully prepared [36].

**Figure 4:**
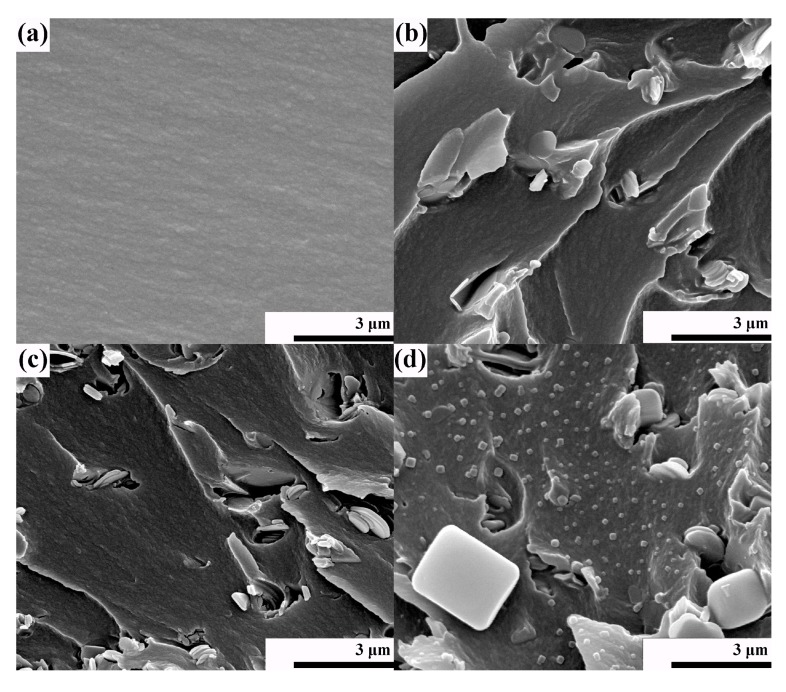
SEM images of PEN-g-BN0 (**a**), PEN-g-BN8 (**b**), PEN-g-BN16 (**c**), and BN/PEN with 8 wt % of BN (**d**, reprinted with permission from [32]).

The dispersivity of BN-CN in the PEN matrix is critical for the properties of the PEN-g-BN hybrids. Therefore, SEM was also employed to characterize the fabrication of the PEN-g-BN hybrids. As shown in Figure 4a, an SEM image of PEN-g-BN0 presents a homogeneous phase with a smooth and compact surface structure. Figure 4b,c are the SEM images of PEN-g-BN8 and PEN-g-BN16, respectively, in which BN-CN is homogeneously dispersed in the PEN matrix without obvious agglomeration. This has mainly resulted from the phthalonitrile modification of BN, which improves the compatibility of BN-CN with the PEN matrix. In addition, when comparing BN/PEN with 8 wt % of BN (Figure 4d), BN-CN adheres to the surface of PEN, and no obvious interface is observed between BN-CN and PEN. This is direct evidence resulting from the cross-linking reaction between the cyano groups on the surface of the BN-CN and from PEN. All the SEM images indicate that the BN-CN can form stable interfacial interactions with PEN matrix, which can potentially increase the performance of the PEN-g-BN hybrids.

**Figure 5:**
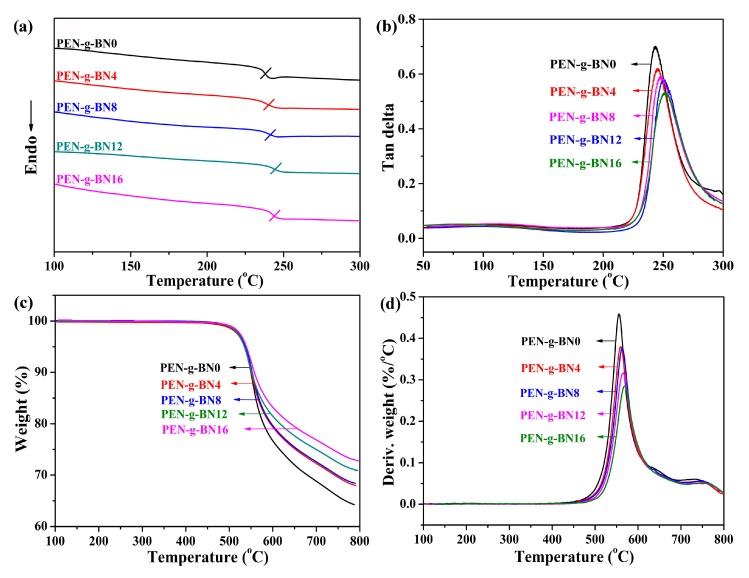
DSC curves (**a**), tan delta curves (**b**), TGA curves (**c**) and DTG curves (**d**) of the PEN-g-BN hybrids.

After the fabrication and characterization of the PEN-g-BN hybrids, their improved properties were investigated in detail. As a high-performance polymer, PEN and its composites demonstrate excellent thermal resistance and mechanical properties. Figure 5 shows the glass transition temperature of the PEN-g-BN hybrids, measured by DSC (Figure 5a) and DMA (Figure 5b). It can be seen from Figure 5a that *T*_g_ of all samples is above 235 °C, suggesting a high thermal resistance property. In addition, the *T*_g_ of the PEN-g-BN hybrids can be seen to increase with increase of the mass fraction of BN-CN (Figure 5a,b). More specifically, the *T*_g_ obtained by DSC is as high as 244 °C for PEN-g-BN16 (Table 1). On one hand, the increasing of *T*_g_ is due to the incorporation of BN as the additives restrict the movement of the PEN main-chains. On the other hand, the crosslinking between BN-CN and PEN also prevents the motion of PEN, thus improving the *T*_g_ of the hybrid. The TGA curves of the PEN-g-BN hybrids are shown in Figure 5c. The decomposition temperatures (*T*_d5%_) of the hybrids are listed in Table 1. The thermal stability of the PEN-g-BN hybrids is also seen to improve with the increasing content of BN-CN, as confirmed by the fact that the peak on the DTG curves of the hybrids gradually shifts to higher temperatures gradually the increasing content of BN-CN (Figure 5d). Undoubtedly, the residual weight of the PEN-g-BN hybrid increases with the increasing of BN-CN.

**Figure 6:**
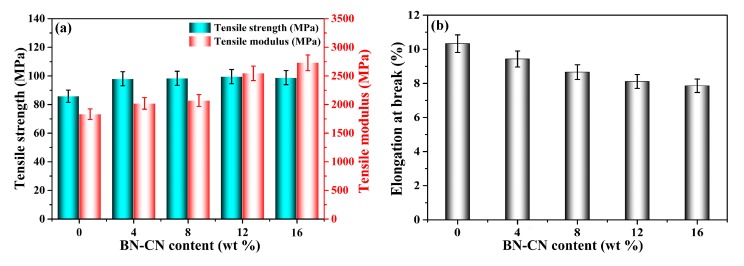
Tensile strength and tensile modus (**a**) and elongation at break (**b**) of the PEN-g-BN hybrids.

Tensile strength, tensile modulus and elongation at break, and the typical mechanical properties of the PEN-g-BN hybrids are shown in Figure 6. It can be seen that all composites exhibit excellent mechanical properties (Table 1). Generally, the tensile strength of composites decreases due to incompatibility between the inorganic fillers and the organic polymer matrix [37]. Additionally, the fillers tend to aggregate at higher content. However, in our system, the tensile strength of the PEN-g-BN hybrids can be found to improve effectively as the BN-CN content increases. In particular, when the BN-CN content is 16%, the mechanical strength of PEN-g-BN16 can be as high as 99 MPa, mainly due to the modification of BN and the formation of a hybrid structure. The tensile modulus is another important parameter which is usually used for the characterization of mechanical properties of a sample. Generally, the tensile modulus can be regarded as an index measuring the difficulty of elastic deformation. The higher the tensile modulus, the more rigid the material. As the BN is much more rigid than PEN, the increase of BN content results in the increase of the tensile modulus of the composites. The tensile modulus of the PEN-g-BN hybrids also shows an increasing trend with the increase of BN-CN content. As for the elongation at break of the PEN-g-BN hybrids, this decreases gradually with the increasing content of BN-CN. This phenomenon can be explained by the increasing cross-linking density of the system. 

**Figure 7:**
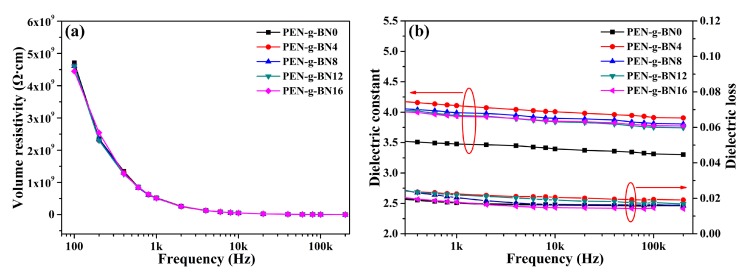
Insulation properties (**a**) and dielectric properties (**b**) of the PEN-g-BN hybrids.

As there are no free electrons in PEN and it shows poor crystallization, PEN demonstrates poor thermal conductivity. As a result, the main purpose of this paper was to improve the thermal conductivity of PEN by introducing fillers into the PEN matrix. However, the addition of fillers may lead to increase of the electrical conductivity of the system, making the resulted composite unsuitable for use in the field of electronic packaging. Therefore, the electrical properties, especially the electrical properties of the PEN-g-BN hybrids at high temperatures, were investigated. Figure 7a shows the volume resistivity of the PEN-g-BN hybrids at different testing frequencies. As a non-conductive polymer, PEN is an insulator with a volume resistivity of 4.71 × 10^9^ Ω∙cm at 100 Hz. It is notable that the addition of BN-CN has little effect on the volume resistivity of the PEN-g-BN hybrids, as BN is also an insulator (Table 1). In addition to this, the dielectric property, another important electrical property for insulators, of the PEN-g-BN hybrids was studied. Figure 7b depicts the dielectric constant and dielectric loss of the PEN-g-BN hybrids. For dielectrics, their permittivity decreases with the increase of frequency due to Maxwell-Wagner polarization. Permittivity of PEN also decreases with frequency, but it exhibits excellent stability with frequency variation, and the frequency coefficient of the dielectric constant is seen to be 1.35 × 10^−6^ Hz^−1^. With the introduction of BN-CN, the dielectric constant of PEN-g-BN hybrids is found to be higher than that of PEN (PEN-g-BN0). However, BN-CN is neither a dielectric material nor a conductive material, and the increase of the dielectric constant of the PEN-g-BN hybrids is mainly caused by the interfacial polarization between the systems [38]. More importantly, although the addition of BN-CN introduces more interfaces, which will lead to increased polarization of the interface, the frequency coefficient of the dielectric constant of the system was found in this work to remain low (Table 1). Another phenomenon which can be seen in Figure 7b is that as the BN-CN content increases, the dielectric constant of the PEN-g-BN hybrids gradually decreases. This is mainly because PEN-g-BN hybrids are a cross-linked system. As filler content increases, crosslinking density of the PEN-g-BN hybrids increases and the number of polar cyano groups decreases, which leads to a decrease in the dielectric constant [39]. The dielectric loss of PEN-g-BN hybrids is similar to that of their dielectric constant. In addition, the value of dielectric loss of PEN-g-BN hybrids is lower than 0.02, which is very important for their practical application.

**Figure 8:**
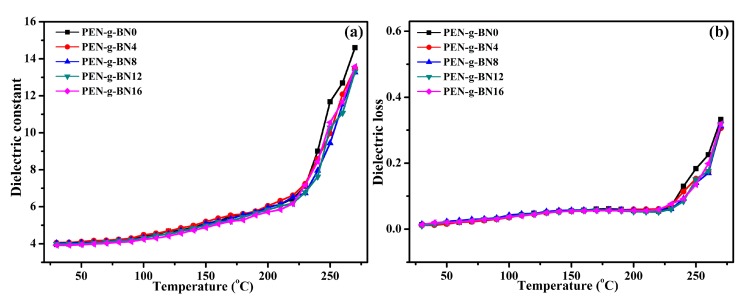
Temperature dependence of dielectric constant (**a**) and dielectric loss (**b**) of the PEN-g-BN hybrids.

The dielectric properties of the PEN-g-BN hybrids at high temperatures were further investigated, as shown in Figure 8. Literature has shown that the dielectric constant of PEN and its composites hardly changes with changing of temperature when the temperature is lower than its *T*_g_. However, when the temperature is close to or higher than its *T*_g_, the dielectric constant increases rapidly [40]. It can be seen that when the testing temperature is lower than 240 °C, the dielectric constant of the PEN-g-BN hybrids seldom changes with increasing testing temperature. However, when the testing temperature is higher than 240 °C, the dielectric constant of the PEN-g-BN hybrids increases sharply. As a result, the *T*_g_ of the hybrids is around 240 °C, according to their dielectric constant. The *T*_g_ obtained from the dielectric constant-temperature curves can be seen to be 233.2, 234.8, 235.5, 236.1, and 236.3 °C for PEN-g-BN0, PEN-g-BN4, PEN-g-BN8, PEN-g-BN12, and PEN-g-BN16 respectively, which are the same values as those measured using DSC. In addition, the similar *T*_g_ of the PEN-g-BN hybrids indicates that the addition of BN-CN has little effect on its thermal properties. The dielectric loss of the PEN-g-BN hybrid materials exhibits a similar phenomenon to that of the dielectric constant with the change in temperature (Figure 8b).

**Figure 9:**
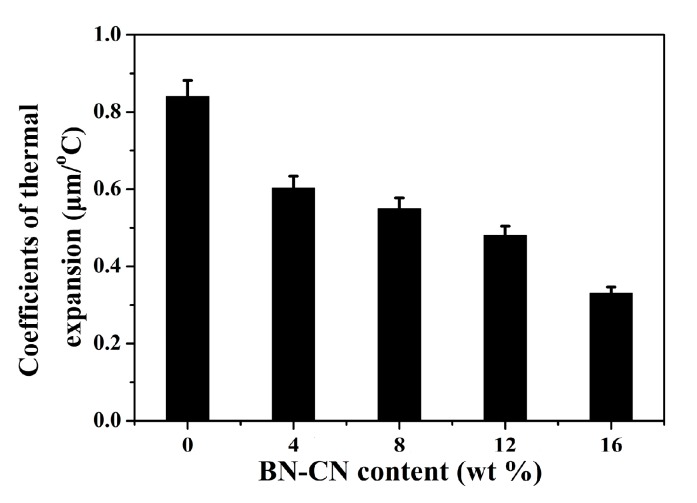
Coefficients of thermal expansion value of the PEN-g-BN hybrids.

Finally, the thermal conductivity of the PEN-g-BN hybrids was investigated. Generally, increasing thermal conductivity of a material allows heat to be quickly conducted, thereby increasing the using temperature of the material. PEN and its composite materials, as high-performance polymer materials, show high a *T*_g_ and thermal decomposition temperature. Hence, PEN materials can be used in high temperature environments. However, if the heat of PEN cannot be transmitted in time when it is used at high temperature, the accumulated heat will increase the local temperature of the material, resulting in its service temperature being lower than its melting point or *T*_g_. Therefore, it is very important to study the dimensional stability of PEN as a function of temperature. Generally, the coefficient of thermal expansion is used to characterize the dimensional stability of materials, and the lower the CET, the better the dimensional stability [41]. The CTE of the PEN-g-BN hybrids was characterized by DMA testing. As shown in Figure 9, the CTE was found to be 0.84 μm/°C for PEN-g-BN0. This low CTE value for PEN-g-BN0 results from the high performance of the PEN-based materials. When the BN-CN is incorporated, CET of the obtained PEN-g-BN hybrids decreases gradually. More specifically, the CET of PEN-g-BN16 is found to be as low as 0.33 μm/°C. The effective reduction of CTE value of composites can be attributed to the good dispersion of BN-CN in the PEN matrix, which hinders the expansion of polymer molecular chains at high temperature. In addition, incorporated BN with excellent thermal conductivity can conduct the heat accumulated in the system in a timely manner, which also depresses the CTE of the obtained hybrid.

**Figure 10:**
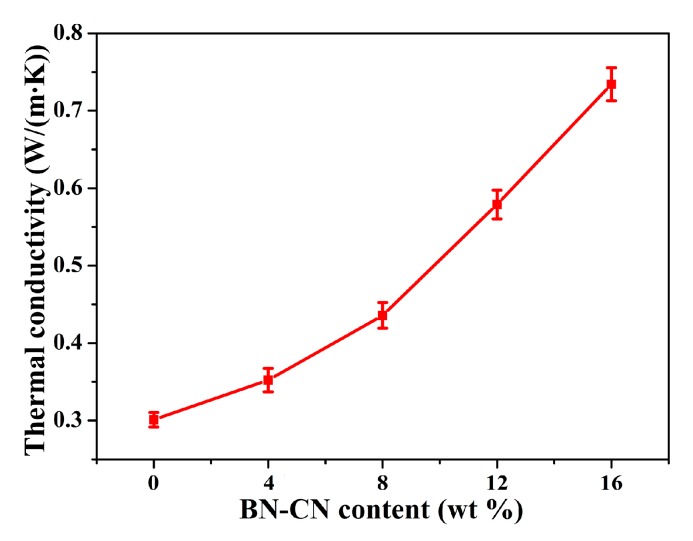
Thermal conductivity of the PEN-g-BN hybrids.

Figure 10 demonstrates the effect of BN-CN content on thermal conductivity of PEN-g-BN hybrids. It can be seen that as the BN-CN content increases, the thermal conductivity of PEN-g-BN hybrids increases obviously. Specifically, for PEN-g-BN0 (PEN), the thermal conductivity is 0.30 W/(m·k). It increases to 0.74 W/(m·k), an increase of 146.7%, when the BN-CN content is 16 wt %. There are two reasons for this. First, the increase in thermal conductivity of the PEN-g-BN hybrids would be caused by the addition of BN-CN, which is a highly conductive channel for heat transportation. The second reason would be that the covalent connection between the BN-CN and PEN substrates promotes heat transformation effectively. This thermal conductivity of PEN-g-BN16 is higher than those of the PEN composites with BNNS and the PEN composites with SPEN-modified BN. In addition, the BN-CN can be easily prepared without any post- treatment after the reaction, even though a two-step procedure is needed. As a result, the high thermal conductivity PEN-g-BN hybrids can be easily and quantitatively produced, which demonstrates their potential application at elevated temperatures.

**Figure 11:**
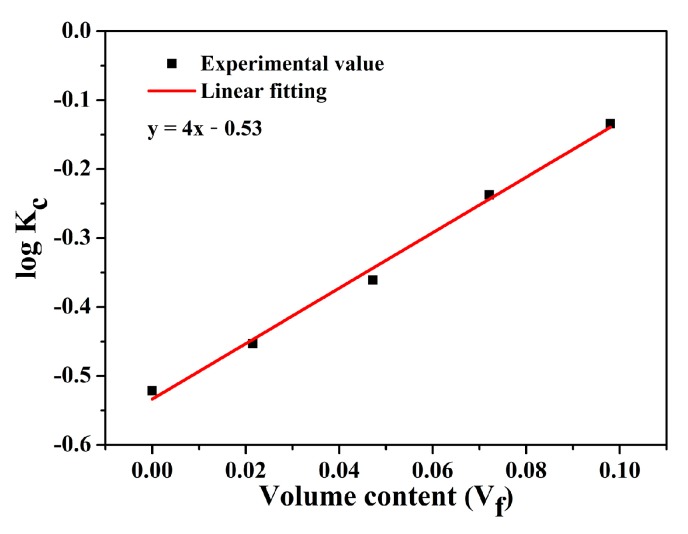
Thermal conductivity of PEN-g-BN hybrids using the Agari model.

In this study, the Agari model [42,43] was employed in further analyzing the thermal conductivity of the hybrids. As shown in Equation (1), effects of dispersion states by introducing factors *C*_1_ and *C*_2_ are considered in the Agari model, i.e.,

log*k*_c_ = *V*_f_*C*_2_log*k*_f_ + (1 − *V*_f_)log(*C*_1_*k*_m_) (1)

where *V*_f_ is the volume fraction of the filler; *k*_c_, *k*_f_, and *k*_m_ are the thermal conductivity of the composite, filler, and matrix, respectively; *C*_1_ is the factor determined by the polymer structure (for example structure, crystallinity or crystal size, etc.); and *C*_2_ is the factor demonstrating the difficulty in formation of the thermal conductivity pathway. In this work, the content of BN-CN is described by mass fraction, and thereby the mass fraction is converted into the volume fraction by Equation (2), i.e.,

*V*_f_ = (*Wρ*_PEN_)/(*Wρ*_PEN_ + (1 − *W*)*ρ*_BN_) (2)

where *W* represents the mass fraction of the filler, *ρ*_PEN_ = 1.3 g/cm^3^, and *ρ*_BN_ = 2.29 g/cm^3^. Figure 11 shows the fitting result of the thermal conductivity of the PEN-g-BN hybrid based on the Agari model. According to the fitting line, the slope and intercept can be obtained, and, hence, *C*_1_ and *C*_2_ are calculated to be 0.98 and 1.41, respectively. Usually, the closer to 1 the value of *C*_2_, the easier it is to form the conductive channels. This high value of *C*_2_ calculated from the result indicates that it is not easy to form conductive channels in the hybrids. As a result, the improved thermal conductivity would be the result of the formation of the hybrids, which promotes heat transformation from BN-CN to PEN. More importantly, thermal conductivity of the PEN-g-BN hybrids can be further improved theoretically as the volume content of BN-CN used in this study is lower than 10 vol %.

## 4. Conclusions

In this study, enhanced thermal conductivity of PEN was achieved by fabricating hybrid materials (PEN-g-BN) through the self-crosslinking of –CN from BN-CN and PEN-Ph. BN-CN were fabricated from BN-OH, IPDI, and 3-APN and then characterized by FT-IR, UV-Vis, and XPS. Following this, BN-CN was incorporated into the PEN-Ph matrix followed by self-crosslinking at a high temperature to obtain PEN-g-BN hybrids. According to the FT-IR, UV-Vis, SEM, gel content, and cross-linking density results, PEN-g-BN hybrids were successfully prepared. The PEN-g-BN hybrids demonstrate excellent thermal resistance with their glass transition temperature and decomposition temperatures being higher than 235 °C and 530 °C, respectively. When 16 wt % BN-CN was added, the obtained hybrid showed a CTE of 0.33 μm/°C and a thermal conductivity of up to 0.74 W/(m·k). This thermal conductivity of PEN-g-BN16 is higher than those of PEN composites with BNNS and PEN composites with SPEN-modified BN. In addition, as PEN-g-BN hybrids can be easily and quantitatively produced, they can show competitiveness for applications at elevated temperatures.

## Figures and Tables

**Table 1:** Gel content, cross-linking density, glass transition temperature obtained by differential scanning calorimetry (DSC) (*T*_g_), glass transition temperature obtained by dynamic mechanical analysis (DMA) (*T*_g_’), thermal decomposition temperature obtained by TGA curve (*T*_d5%_), thermal decomposition temperature obtained by DTG curve (*T*_d_), tensile strength, tensile modulus, elongation at break, volume resistivity at 100 Hz, and frequency coefficient of the dielectric constant of the PEN-g-BN hybrids.

Sample	PEN-g-BN0	PEN-g-BN4	PEN-g-BN8	PEN-g-BN12	PEN-g-BN16
Gel content (%)	88.9	90.2	91.4	93.9	95.1
Cross-linking density (mol/cm^3^)	0.576	0.635	0.744	0.795	0.801
*T*_g_ (°C)	238.05	240.25	241.30	243.75	244.05
*T*_g_’ (°C)	243.1	245.3	248.5	250.3	251.2
*T*_d5%_ (°C)	530.3	532.4	534.7	535.9	536.7
*T*_d_ (°C)	555.4	559.6	562.2	564.7	567.9
Tensile strength (MPa)	85.85	98.04	98.38	99.54	99.83
Tensile modulus (GPa)	1.83	2.02	2.07	2.55	2.73
Elongation at break (%)	10.4	9.5	8.7	8.1	7.9
Volume resistivity (Ω∙cm)	4.71 × 10^9^	4.61 × 10^9^	4.61 × 10^9^	4.60 × 10^9^	4.45 × 10^9^
Frequency coefficient of dielectric constant (Hz^−1^)	1.35 × 10^−6^	1.65 × 10^−6^	1.45 × 10^−6^	1.60 × 10^−6^	1.30 × 10^−6^
